# Phase II study of RC-160 (vapreotide), an octapeptide analogue of somatostatin, in the treatment of metastatic breast cancer

**DOI:** 10.1038/sj.bjc.6690226

**Published:** 1999-03

**Authors:** K J O'Byrne, N Dobbs, D J Propper, J P Braybrooke, M I Koukourakis, K Mitchell, J Woodhull, D C Talbot, A V Schally, A L Harris

**Affiliations:** Imperial Cancer Research Fund Medical Oncology Unit, Churchill Hospital, Oxford, OX3 7LJ, UK; Endocrine, Polypeptide and Cancer Institute, Veterans Affairs Medical Centre, and Department of Medicine, Tulane University Medical School, New Orleans, LA, LA 70112, USA

**Keywords:** somatostatin, RC-160, breast cancer, metastatic, insulin-like growth factor-I, prolactin

## Abstract

RC-160 (octastatin/vapreotide) is a potent octapeptide analogue of somatostatin with growth inhibitory activity in experimental tumours in vitro and in vivo, including breast cancer. We evaluated the efficacy and tolerability of high-dose RC-160, 3 mg day^−1^ on week 1 increased to 4.5 mg day^−1^ for weeks 2–4 and subsequently 6 mg day^−1^ until the end of treatment, administered by continuous subcutaneous infusion in the management of 14 women with previously treated metastatic breast cancer. The age range was 37–80 years (median 58.5 years) and performance status 0–2. The treatment was well tolerated with no dose reductions being required. No grade 3 or 4 toxicities were seen. Abscess formation developed at the infusion site in eight patients and erythema and discomfort was seen in a further three patients. A significant reduction in IGF-I levels occurred by day 7 and was maintained throughout the treatment. The lowest dose of RC-160 produced the maximal IGF-I response. Although there was no reduction in prolactin levels in patients whose baseline levels were normal, elevated prolactin levels found in three patients fell to within the normal range 7 days after commencing RC-160 treatment. A small but significant rise in fasting blood glucose levels was also recorded, the highest level on treatment being 7.6 mmol l^−1^. No objective tumour responses were observed, all patients showing disease progression within 3 months of commencing treatment. These findings demonstrate that high-dose RC-160, administered as a continuous subcutaneous infusion, can reduce serum levels of the breast growth factors IGF-I and prolactin but is ineffective in the management of metastatic breast cancer. Encouraging preclinical anti-tumour activity and the favourable toxicity profile in patients suggest the merit of future studies combining RC-160 with anti-oestrogen, cytotoxic and anti-angiogenic agents. © 1999 Cancer Research Campaign


*British Journal of Cancer* (1999) **79**(9/10), 1413–1418
© 1999 Cancer Research Campaign
Article no. bjoc.1998.0226

### Phase II study of RC-160 (vapreotide), an octapeptide analogue of somatostatin, in the treatment of metastatic breast cancer

**KJ O'Byrne[1], N Dobbs[1], DJ Propper[1], JP Braybrooke[1], MI Koukourakis[1], K Mitchell[1], J Woodhull[1], DC Talbot[1], AV Schally[2] and AL Harris[1]**

[1]Imperial Cancer Research Fund Medical Oncology Unit, Churchill Hospital, Oxford OX3 7LJ, UK; [2]Endocrine, Polypeptide and Cancer Institute, Veterans Affairs Medical Centre, and Department of Medicine, Tulane University Medical School, New Orleans, LA 70112, USA

**Summary** RC-160 (octastatin/vapreotide) is a potent octapeptide analogue of somatostatin with growth inhibitory activity in experimental tumours in vitro and in vivo, including breast cancer. We evaluated the efficacy and tolerability of high-dose RC-160, 3 mg day$^{-1}$ on week 1 increased to 4.5 mg day$^{-1}$ for weeks 2–4 and subsequently 6 mg day$^{-1}$ until the end of treatment, administered by continuous subcutaneous infusion in the management of 14 women with previously treated metastatic breast cancer. The age range was 37–80 years (median 58.5 years) and performance status 0–2. The treatment was well tolerated with no dose reductions being required. No grade 3 or 4 toxicities were seen. Abscess formation developed at the infusion site in eight patients and erythema and discomfort was seen in a further three patients. A significant reduction in IGF-I levels occurred by day 7 and was maintained throughout the treatment. The lowest dose of RC-160 produced the maximal IGF-I response. Although there was no reduction in prolactin levels in patients whose baseline levels were normal, elevated prolactin levels found in three patients fell to within the normal range 7 days after commencing RC-160 treatment. A small but significant rise in fasting blood glucose levels was also recorded, the highest level on treatment being 7.6 mmol l$^{-1}$. No objective tumour responses were observed, all patients showing disease progression within 3 months of commencing treatment. These findings demonstrate that high-dose RC-160, administered as a continuous subcutaneous infusion, can reduce serum levels of the breast growth factors IGF-I and prolactin but is ineffective in the management of metastatic breast cancer. Encouraging preclinical anti-tumour activity and the favourable toxicity profile in patients suggest the merit of future studies combining RC-160 with anti-oestrogen, cytotoxic and anti-angiogenic agents.

**Keywords:** somatostatin; RC-160; breast cancer; metastatic; insulin-like growth factor-I; prolactin

Somatostatin is a tetradecapeptide hormone first identified in the hypothalamus as an inhibitor of growth hormone (GH) release. The peptide has subsequently been found throughout the body, particularly in the pancreas, gastrointestinal tract and nervous system. As well as inhibiting hormone release, somatostatin functions as a neurotransmitter, immunomodulator and suppressor of angiogenesis and cell proliferation (Reichlin, 1983*a,b*; Schally, 1988; Patel et al, 1994; Pollak and Schally, 1998). Somatostatin acts by binding to specific receptors (sst) of which five principal subtypes have been identified: sst1, sst2, sst3, sst4 and sst5 (Bruns et al, 1994).

RC-160 (D-Phe-Cys-Tyr-D-Trp-Lys-Val-Cys-Trp-$NH_2$) is a potent cyclic octapeptide analogue of somatostatin (Cai et al, 1986). RC-160 is up to 500 times more potent than octreotide and somatuline in inhibiting the synthesis/release of hormones from sst expressing pituitary and malignant neuroendocrine cells, although the maximal inhibitory effects are similar (Hofland et al, 1994). RC-160 inhibits the growth of sst positive colonic, pancreatic, gastric, lung and both androgen-dependent and -independent prostatic cancer and glioblastoma and osteosarcoma tumours (Schally, 1988; Qin et al, 1995; Pinski et al, 1996; Pollak and Schally, 1998). In transfection experiments, sst2 and sst5 have been shown to be the principal receptor subtypes involved in mediating the growth inhibitory effects of RC-160 (Buscail et al, 1994, 1995; Cordelier et al, 1997). RC-160 activates phosphotyrosine phosphatases (PTPs), which dephosphorylate tyrosine residues of activated type 1 growth factor receptors, including epidermal growth factor, and inhibits intracellular cAMP and cGMP accumulation and calcium mobilization (Schally, 1988; Buscail et al, 1994; Buscail et al, 1995; Qin et al, 1995; Cordelier et al, 1997; Pollak and Schally, 1998). The growth inhibitory effects of RC-160 in sst positive cancer cell lines have been directly linked to activation of PTPs and inhibition of intracellular cAMP production in vitro (Liebow et al, 1988; Qin et al, 1995).

Somatostatin analogues also inhibit the growth of sst negative tumours in in vivo studies, suggesting important indirect anti-proliferative effects. Many tumours express insulin-like growth factor-I (IGF-I) receptors and proliferate in response to exposure to IGF-I (Macaulay, 1992). Through down-regulation of the growth hormone GH/IGF-I axis, somatostatin analogues may inhibit the growth of IGF-I responsive tumours (Holly, 1998; Pollak and Schally, 1998). The growth inhibitory effects of RC-160 in sst negative tumours are associated with a decrease in serum growth hormone (GH) and IGF-I levels and in tumour IGF-I receptor expression (Pinski et al, 1994, 1996). Somatostatin analogues are also potent anti-angiogenic agents, having direct growth inhibitory effects on proliferating endothelial cells (Patel et

*Received 19 January 1998*
*Revised 3 September 1998*
*Accepted 9 October 1998*

*Correspondence to:* J O'Byrne, Consultant and Senior Lecturer in Medical Oncology, Osborne Building, Leicester Royal Infirmary, Leicester LE1 5WW, UK

al, 1994). As angiogenesis is essential for tumour growth beyond 1–2 mm in diameter, inhibition of this process may result in tumour growth inhibition (Folkman, 1995).

In the region of between 70 and 90% of breast tumours express specific high affinity binding sites for radiolabelled somatostatin analogues (Prevost and Israel, 1993, Van Eijck et al, 1994). The principal subtype detected is sst2 (Evans et al, 1997). The octapeptide somatostatin analogues, including RC-160, inhibit the growth of sst expressing breast tumours in vitro and in vivo (Setyano-Han et al, 1987; Szende et al, 1989; Szende et al, 1991; Brower et al, 1992; Szepeshazi et al, 1992; Prevost and Israel, 1993). The regressive changes in vivo are consistent with apoptosis and coagulation necrosis (Szende et al, 1989, 1991). IGF-I is a potent mitogen for breast cancer cells. The growth effects are mediated through high affinity IGF-I receptors, which have been found to be expressed by the majority of breast tumours (Klijn et al, 1993; Helle and Lonning, 1996). RC-160 therapy results in down-regulation of IGF-I receptor expression in MXT mouse mammary carcinoma (Srkalovic et al, 1989). Therefore, RC-160 therapy may inhibit breast cancer growth through modulation of the mitogenic effects of IGF-I.

A number of small studies have evaluated somatostatin analogues in the management of breast cancer. Treatment rarely induces an objective response, although disease stabilization has been reported in 20–43% of cases. When studied, only a moderate and non-durable reduction in IGF-I levels was seen (Manni et al, 1989; Morere et al, 1989; Vennin et al, 1989; Stolfi et al, 1989; Prevost and Israel, 1993; Di Leo et al, 1995; Ingle et al, 1996). Toxicity in all studies was mild, transient diarrhoea being the most common observed problem. Other side-effects of somatostatin analogue therapy include pain at the injection site, glucose intolerance and gallstone formation (Battershill and Clissold, 1989).

The work presented evaluated single agent, high-dose, continuous infusional RC-160 in the management of patients with pretreated metastatic breast cancer.

### PATIENTS AND METHODS

### Entry criteria

In a phase II single-centre study we evaluated the efficacy of RC-160 (Debiopharm SA, Lausanne, Switzerland) in the management of patients with relapsed breast cancer following previous systemic therapy. Inclusion criteria included cytologically and/or histologically proven breast cancer, at least unidimensionally measurable disease, age ≥ 18 years, life expectancy ≥ 3 months, performance status ≤ 2, haemoglobin ≥ 10 g $dl^{-1}$, white cell count ≥ 3 × $10^9$ $l^{-1}$, absolute neutrophil count ≥ 2 × $10^9$ $l^{-1}$, platelets ≥ 100 × $10^9$ $l^{-1}$, bilirubin ≤ 2 × normal (N), aspartate and alanine transferase ≤ 3 × N unless due to metastases where values ≤ 5 × N were accepted and plasma creatinine ≤ 150 μmol $L^{-1}$. Patients who had received intensive chemotherapy or radiotherapy within the previous 3 weeks, pregnant women or, where appropriate, those not taking adequate contraceptive precautions were excluded from the study.

Pretreatment evaluation included a minimum of a history and physical examination, a full blood count (FBC), renal, liver and bone biochemistry, fasting blood sugar, a chest X-ray and ultrasound examination of the abdomen. Response was assessed according to standard WHO criteria. All evaluable/measurable sites of disease were recorded prior to commencing therapy employing further radiological techniques as deemed necessary. Clinical photographs and measurements of skin lesions and/or lymph nodes were also performed and utilized for response assessment where appropriate.

The RC-160 was supplied as a lyophilized powder in sterile vials. The powder was dissolved in sterile water for injection. The powder contained a glutamate excipient and the reconstituted solution had a pH = 4.5 (Debiopharm SA). The reconstituted solution was administered by continuous subcutaneous infusion using a Walkmed 350 pump (Medex Medical Inc., Rossendale, UK). Continuous infusion was chosen to facilitate the administration of high-dose RC-160, to maintain relatively steady-state RC-160 plasma levels (not formally tested) and because RC-160 is being developed as a slow release formulation for depot injection (unpublished data), as have the other principal somatostatin analogues octreotide and somatuline (Caron et al, 1997; Helle et al, 1998). Initially, either a 23G Butterfly needle (Venisystems, Sligo, Ireland) or a 22G Intima catheter (Becton Dickinson, Sandy, Utah, USA) was implanted into the anterior abdominal wall, alternating between the two types for each patient to investigate the efficacy and side-effects of each. The RC-160 infusion was started at 3 mg $day^{-1}$ increasing after 1 week to 4.5 mg $day^{-1}$ and after 4 weeks to 6 mg $day^{-1}$. Patients were treated on an in-patient basis for the first 2 days of therapy in order to become familiar with the pumps and to report any untoward side-effects. No problems were observed with RC-160 solubility in preclinical testing, at the time of preparation of the solution or during treatment.

Planned follow-up assessments included an FBC, biochemistry profile and fasting blood sugar, IGF-1 and PRL levels on days 7, 28 and monthly thereafter. Measurable sites of disease were to be evaluated after 28 days and subsequently every 2 months. The study was approved by the local Ethics Committee and patients were included only after giving informed written consent.

### Serum insulin-like growth factor I and prolactin levels

IGF-I and PRL levels were evaluated in fasting serum samples obtained prior to commencing RC-160 treatment, and after 1, 4 and every 4 weeks thereafter until treatment was discontinued. IGF-I levels were measured using the Octeia IGF-I kit, a two site immunoenzymometric assay (IEMA) (Immunodiagnostic Systems Ltd., Boldon, Tyne and Wear, UK). The sensitivity of the assay is < 1 nmol $l^{-1}$. The intra-assay coefficient of variance is 2.3–3.5% and the interassay variance is 6.95–7.14%. The normal ranges are 9.5–45 nmol $l^{-1}$ for women aged 21–40 years, 7.5–30 nmol $l^{-1}$ for women aged 41–60 years and 5–22.5 nmol $l^{-1}$ for women aged > 60 years. PRL levels were measured using AxSYM prolactin, a microparticle enzyme immunoassay (MEIA) (Abbott Laboratories, Diagnostics Division, Abbott Park, IL 60064, USA). The sensitivity of the assay is 15 mU/$l^{-1}$. The intra-assay coefficient of variance is 2.81–4.13% and the interassay variance is 0–4.97%. The normal range is 33–580 mU/$l^{-1}$.

### Statistical methods

The paired Student's *t* test was used to determine the equality of paired means for glucose, IGF-I and PRL levels on treatment. The analysis was performed employing the Stata statistical software, release 5.0 package (Stata, College Station, TX, USA).

© Cancer Research Campaign 1999

**Table 1** Clinical characteristics of patients (pts)

| | |
|---|---|
| Fourteen women recruited to study | |
| Age: median | 61.3 years |
| range | 37–80 years |
| ECOG performance status | 0–2 (median 1) |
| *Histological subtypes* | |
| Invasive ductal | 10 pts |
| Lobular | 2 pts |
| Mixed ductal and lobular | 1 pt |
| Other | 1 pt |
| *Previous treatments* | |
| Prior endocrine therapy | 13 pts |
| adjuvant | 6 pts |
| advanced | 10 pts |
| Prior chemotherapy | 12 pts |
| adjuvant | 7 pts |
| advanced | 10 pts |
| Prior radiotherapy | |
| local | 14 pts |
| ablative oophorectomy | 1 pts |
| metastatic | 3 pts |
| *Sites of disease* | |
| Skin | 8 pts |
| Lymph nodes | 6 pts |
| Bone | 5 pts |
| Pleura | 5 pts |
| Soft tissue | 3 pts |
| Lung | 3 pts |
| Liver | 2 pts |
| Breast | 2 pts |
| Adrenal | 1 pt |

**Table 2** Number of patients developing grade 1 and 2 toxicities based on CALGB common toxicity criteria (total number of patients in study = 14)

| | Grade | |
|---|---|---|
| **Toxicity** | **1** | **2** |
| Diarrhoea | 9 | 3 |
| Vomiting | 4 | 1 |
| Nausea | 4 | 3 |
| Fatigue | 7 | 5 |
| Headache | 6 | 1 |
| Constipation | 2 | 1 |
| Abdominal pain | 3 | 2 |
| Inflammation at infusion site | 7 | 4 |

No grade 3 or 4 toxicities were seen in any of the patients. Four patients with inflammation at the infusion site were treated with antibiotics until we determined that the problem was due to sterile abscess formation. Borborygmi were recorded in two patients and steatorrhoea in five. Haematological toxicities were rare with two patients developing grade 1 neutropenia and one a grade 2 leucopenia.

### RESULTS

Fourteen women, age range 37–80 years (median 58.5 years), ECOG performance status 0–2, with stage IV breast cancer were recruited to the study. Patient characteristics are summarized in Table 1. All patients had received prior radiotherapy and endocrine agents and/or cytotoxic chemotherapy on at least one occasion, either in the adjuvant setting or for advanced disease.

### Toxicity

Toxicities are summarized in Table 2. No grade 3 or 4 toxicities were seen. Haematological toxicities were virtually absent, while no alterations were seen in renal or liver function. A significant increase was seen in fasting blood glucose levels, the mean rising from 5.3 mmol $l^{-1}$ to 6.2 mmol $l^{-1}$ ($P = 0.0012$). The most common side-effects included mild fatigue and diarrhoea. The diarrhoea was associated with steatorrhoea in five cases. Two patients reported a marked increase in borborygmi. Regular anti-emetics were required for mild nausea by seven patients. Both patients with liver metastases experienced nausea which, although present at baseline, worsened on treatment

The most troublesome side-effect was inflammation at the infusion site seen in 11 patients associated with the development of subcutaneous abscesses in eight. Two cases were lanced. These and two others were treated with oral antibiotics. In two cases swab cultures grew *Staphlococcus aureas*. However, it became apparent with time that the abscesses probably represented local reactions either to the cannulas or to the RC-160 infusion itself. This led to a policy of resiting the cannula site weekly, whether or not there was a problem. Subsequently, abscess formation became less of a problem without the need for therapeutic intervention. Regarding the assessment of cannula type, the Intima cannula proved more user friendly than Butterfly needles, being subjectively more comfortable for the patient and easier to secure.

### Serum IGF-I and PRL levels

IGF-I levels fell in all patients treated with RC-160. The mean level for the 14 patients decreased by 45% by day 7 ($P < 0.0001$). No subsequent significant change was seen between days 7, 28 and 56, despite increasing doses of the medication (Figure 1). Only three patients were sampled on day 84. Again the values were similar to those on day 7. Samples were obtained from two patients off treatment and doubled from the last on treatment sample. PRL levels were within the normal range in 11 of the 14 patients evaluated and

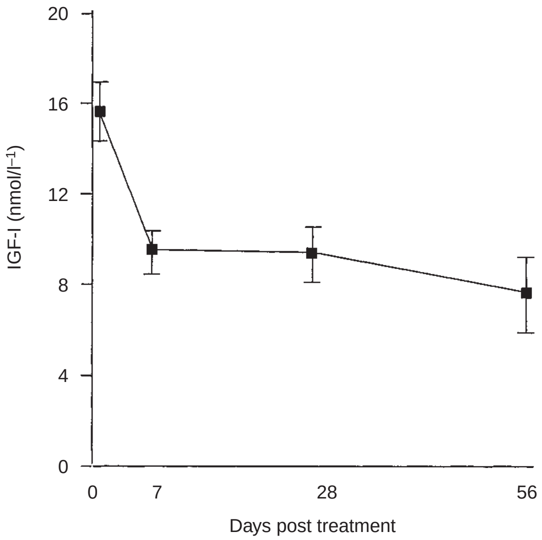

**Figure 1** Serum IGF-1 (mean±SE). Paired test: pre vs. day 7, $P < 0.00001$ ($n = 14$); pre vs. day 28, $P < 0.0002$ ($n = 11$); pre vs. day 56, $P < 0.0009$ ($n = 8$); day 7 vs. day 28, $P$ = NS ($n = 11$); day 28 vs. day 56, $P$ = NS ($n = 8$)

© Cancer Research Campaign 1999

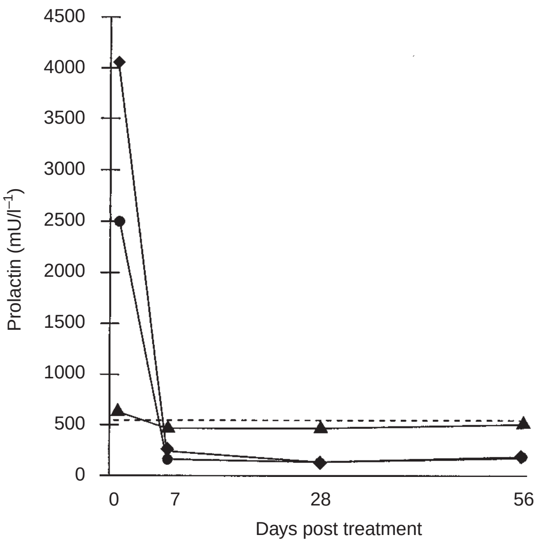

**Figure 2** RC-160 therapy resulted in normalization of serum prolactin in all 3 patients with elevated levels pretreatment were not affected by RC-160 therapy. However, elevated levels seen in three patients fell to normal within 7 days of commencing the RC-160 infusion (Figure 2).

### Response

No objective tumour responses were seen, all patients showing objective evidence of progressive disease within 3 months of commencing RC-160 treatment.

### DISCUSSION

Breast cancer is the most prevalent malignant disease among women in developed countries. When the disease has spread beyond the breast and axilla, it is essentially incurable and therapy is palliative, being directed at prolonging survival and improving symptom control (Hayes et al, 1995; Goldhirsch and Gelber, 1996). Somatostatin analogues are among the novel hormonal agents being investigated for the treatment of breast cancer.

Although RC-160 represents the most potent of the principal somatostatin analogues in clinical development and is well tolerated when administered in high dose as a continuous subcutaneous infusion, the agent proved to be ineffective in the treatment of relapsed, previously treated, metastatic breast cancer. This is in keeping with the results reported for the somatostatin analogues, octreotide and somatuline (Manni et al, 1989; Morere et al, 1989; Stolfi et al, 1989; Vennin et al, 1989; Prevost and Israel, 1993; Di Leo et al, 1995; Ingle et al, 1996). Similar results are also seen in inoperable pancreatic cancer where, although tumour growth was stabilized and symptoms improved in a proportion of patients, it was felt that RC-160 alone, even in large doses, is insufficient to produce effective palliation in these patients (Poston and Schally, 1993).

One of the principal side-effects seen was the development of skin inflammation in 11 patients, associated with cutaneous abscesses at the cannulation site in eight. These were initially treated as being infected. However, with experience the suspicion increased that the abscesses were most probably sterile and represented a reaction to either the cannula or to the infusion itself. Similar findings have been documented in patients treated at other centres with infusional RC-160. In a number of cases the material from the abscesses has been studied microscopically, revealing florid chronic inflammation with infiltration by multinucleated giant cells. In a study in the Yucatan minipig, prolonged subcutaneous catheter implantation induced focal ulceration in the epidermis in one of six animals, fibroblast proliferation in the dermis and local inflammation centred around the catheter. Vapreotide with or without glutamate excipient at a concentration of 1.5 mg ml$^{-1}$ induced granulomas surrounded with fibrosis, associated sometimes with local oedema (Debiopharm SA; unpublished data). This led to a policy of resiting the cannula on a weekly basis, which eased the problem considerably. A small but significant increase in fasting blood glucose levels, and mild gastrointestinal side-effects were also seen in keeping with known side-effects of somatostatin analogue therapy (Battershill and Clissold, 1989). The only therapeutic intervention required was the use of anti-emetics in seven patients.

A significant reduction in serum IGF-I levels was seen, levels falling in all patients as compared with baseline. Unlike previous studies in breast cancer, where lower doses of octreotide and somatuline were used, the high dose continuous infusion of RC-160 induced a 45% reduction in serum IGF-I levels which was sustained over time (Manni et al, 1989: Vennin et al, 1989; Di Leo et al, 1995). This is likely to be a result of the dose used as in a recent phase I study in 14 patients with gastrointestinal tract and pancreatic cancer, microencapsulated octreotide pamoate 90 mg i.m. every 4 weeks or 160 mg i.m. every 2 weeks (> 3 mg day$^{-1}$) produced an equivalent sustained reduction in plasma IGF-I levels of 49–53% (Helle et al, 1998).

The lack of efficacy of single agent somatostatin analogue therapy in breast cancer may in part be due to the antagonistic effects of oestrogen (Setyano-Han et al, 1987). Recent in vitro and in vivo studies indicate that anti-oestrogens may potentiate the anti-tumour activity of somatostatin analogues in breast cancer (Weckbecker et al, 1994; Candi et al, 1995; Pollak, 1996; Xu et al, 1996). In 33 post-menopausal women with untreated breast cancer the combination of depot injections of somatuline with oral tamoxifen resulted in an objective response rate of 50% (95% CI: 35–69%) (Canobbio et al, 1995). The potential therapeutic benefits of combined manipulation of the GH/IGF-I and gonadotropin/sex steroid axes in sex hormone-associated tumours is supported by a recent study combining anti-androgen therapy with RC-160 in 19 men with hormone refractory prostate cancer. After 3 months, 14 patients showed a decrease in serum prostate-specific antigen levels, reduction in bone pain and improvement in Karnofsky performance status (Gonzalez-Barcena et al, 1998).

Experimental evidence suggests that PRL, a growth factor for normal breast tissue, is an autocrine factor for breast cancer. Tumour cells have been demonstrated to express both PRL and PRL-receptors (Clevenger et al, 1995; Das and Vonderhaar, 1996). The prolactin receptor, of which three subtypes have been identified, belongs to the superfamily of cytokine receptors. Binding of PRL to the PRL-receptor results in activation of cytoplasmic signal transducers and transcriptional activators through phosphorylation of JAK2 kinases. Recent work has shown that activation of breast cancer cell line PRL receptors results in stimulation of the *ras-raf*-MEK(MAP kinase kinase)-MAP kinase mitogenic pathway. The activation of ras is mediated via tyrosine phosphorylation of SHC, recruitment of GRB2 and the guanine nucleotide exchange factor SOS (Erwin et al, 1995; Das and Vonderhaar, 1996).

 *© Cancer Research Campaign 1999*

RC-160 reduced serum PRL to within the normal range in all three patients with elevated levels prior to commencing treatment. Somatostatin analogues have little or no effect on primary pituitary hyperprolactinaemia (Lamberts et al, 1991). These findings suggest that the elevated PRL levels seen in the patients in our study are due to synthesis and release of the peptide from breast cancer cells. The reduction of elevated PRL levels to within the normal range and activation of PTPs in tumours expressing sst2 suggest that RC-160 may counteract the tumour growth stimulating effects of PRL in some breast cancer patients. A recent study of triple therapy with tamoxifen, the anti-prolactin agent CV 205–502 and octreotide gave an objective response in five of nine evaluable patients. A significant reduction in IGF-I levels was seen similar to that documented in the present study. Furthermore, a strong significant reduction in circulating PRL levels was documented in patients with normal PRL secretion (Bontenbal et al, 1998). RC-160 may have a role to play in this setting.

In experimental models octreotide has been shown to enhance the efficacy of cytotoxic agents in the treatment of solid tumours while ameliorating their side-effects. The drugs studied include doxorubicin, paclitaxel, mitomycin C and 5-fluorouracil all of which are routinely used in the management of metastatic breast cancer (Lee et al, 1993; Weckbecker et al, 1996). The enhanced anti-tumour activity may in part be explained by the inhibitory effects of somatostatin analogues on angiogenesis, as known anti-angiogenic agents such as TNP-470 and minocycline have been shown to increase the anti-tumour activity of cyclophosphamide (Patel et al, 1994; Teicher et al, 1994). In keeping with these findings, RC-160 has been shown to increase the effectiveness of 5-fluorouracil in vivo (Szepeshazi et al, 1991). These results suggest the merit of evaluating the combination of somatostatin analogues with cytotoxic agents in breast cancer therapy, initially in animal models and subsequently in phase I studies. Cytotoxic analogues of somatostatin containing potent anthracyclines have recently been developed and have shown promising anti-tumour activity in vitro and in vivo in a number of sst positive solid tumours including breast cancer. The possibility of specifically targeting sst-rich tumours in patients with such agents is an exciting prospect for the future (Nagy et al, 1998).

IGF-I is a potent trophic and survival factor for many normal cells including those of the breast and prostate gland. Overexpression of growth hormone and IGF-I receptor agonists is associated with the development of breast cancer in transgenic mice (Tornell et al, 1992; Bates et al, 1995). In two recently published prospective studies, high normal IGF-I levels were associated with an increased relative risk for the development of prostate cancer in men (4.3) and breast cancer in premenopausal women (7.28 in premenopausal women aged < 50 years, when adjusted for IGF binding protein-3 levels) (Chan et al, 1998; Haankinson et al, 1998). Given the sustained suppression of IGF-I levels documented in the present study, RC-160 alone or in combination with tamoxifen or LHRH analogues or antagonists may have a role in the chemoprevention of breast and prostate cancer (Holly, 1998).

In conclusion, although ineffective as single agent therapy, high-dose RC-160 is well tolerated, induces a significant and sustained reduction in serum IGF-1 levels and normalizes hyperprolactinaemia in patients with pretreated metastatic breast cancer. Based on the favourable toxicity profile and the encouraging preclinical findings, further experimental and clinical studies combining RC-160 with anti-oestrogens, antiprolactins, cytotoxic and anti-angiogenic agents are warranted.

### ACKNOWLEDGEMENTS

This work was supported by the Imperial Cancer Research Fund. We would like to thank Debiopharm SA for their generous gift of the RC-160 with which to conduct the study.

### REFERENCES

Bates P, Fisher R, Ward A, Richardson L, Hill DJ and Graham CF (1995) Mammary cancer in transgenic mice expressing insulin-like growth factor II (IGF II). *Br J Cancer* **72**: 1189–1193

Battersill PE and Clissold SP (1989) Octreotide: A review of its pharmacodynamic and pharmacokinetic properties, and therapeutic potential in conditions associated with excessive peptide secretion. *Drugs* **38**: 658–702

Bontenbal M, Foekens JA, Lamberts SWJ, de Jong FH, van Putten WLJ, Braun HJ, Burghouts JThM, van der Linden GHM and Klign JGM (1998) Feasibility, endocrine and anti-tumour effects of a triple endocrine therapy with tamoxifen, a somatostatin analogue and an antiprolactin in post-menopausal metastatic breast cancer: a randomised study with long-term follow-up. *Br J Cancer* **77**: 115–122

Brower ST, Schally AV, Redding TW and Hollander VP (1992) Differential effects of LHRH and somatostatin analogs on human breast cancer. *J Surg Res* **52**: 6–14

Bruns C, Weckbecker G, Raulf F, Kaupmann K, Schoesster P, Hoyer D and Lubbert H (1994) Molecular pharmacology of somatostatin receptor subtypes. *Ann NY Acad Sci* **733**: 138–146

Buscail L, Delesque N, Esteve J-P, Saint-Laurent N, Prats H, Clerc P, Robberecht P, Bell GI, Liebow C, Schally AV, Vaysse N and Susini C (1994) Stimulation of tyrosine phosphatase and inhibition of cell proliferation by somatostatin analogues: mediation by human somatostatin receptor subtypes SSTRI and SSTR2. *Proc Natl Acad Sci USA* **91**: 2315–2319

Buscail L, Esteve J-P, Saint-Laurent N, Bertrand V, Reisine T, O'Carroll A-M, Bell GI, Schally AV, Vaysse N and Susini C (1995) Inhibition of cell proliferation by the somatostatin analogue RC-160 is mediated by somatostatin receptor subtypes SSTR2 and SSTR5 through different mechanisms. *Proc Natl Acad Sci USA* **92**: 1580–1584

Cai R-Z, Szoye B, Lu R, Fu D, Redding TW and Schally AV (1986) Synthesis and biological activity of highly potent octapeptide analogs of somatostatin. *Proc Natl Acad Sci USA* **83**: 1896–1900

Candi E, Melino G, De Laurenzi V, Piacentini M, Guerrieri P, Spinedi A and Knight RA (1995) Tamoxifen and somatostatin affect tumours by inducing apoptosis. *Cancer Lett* **96**: 141–143

Canobbio L, Cannata D, Miglietta L and Boccardo F (1995) Somatuline (BIM 23014) and tamoxifen treatment of postmenopausal breast cancer patients: clinical activity and effect on insulin-like growth factor-I (IGF-I) levels. *Anticancer Res* **15**: 2687–2690

Caron P, Morange-Ramos I, Cogne M and Jaquet P (1997) Three year follow-up of acromegalic patients treated with intramuscular slow-release lanreotide. *J Clin Endocrinol Metab* **82**: 18–22

Chan JM, Stampfer MJ, Giovannucci E, Gann PH, Ma J, Wilkinson P, Hennekens CH and Pollak M (1998) Plasma insulin-like growth factor-I and prostate cancer risk: A prospective study. *Science* **279**: 563–566

Clevenger CV, Chang WP, Ngo W, Pasha TL, Montone KT and Tomaszewski JE (1995) Expression of prolactin and prolactin receptor in human breast carcinoma. Evidence for an autocrine/paracrine loop. *Am J Path* **146**: 695–705

Cordelier P, Esteve J-P, Bousquet C, Delesque N, O'Carroll A-M, Schally AV, Vaysse N, Susini C and Buscail L (1997) Characterization of the anti-proliferative signal mediated by the somatostatin receptor subtypes sst5. *Proc Natl Acad Sci USA* **94**: 9343–9348

Das R and Vonderhaar BK (1996) Activation of raf-1, MEK, and MAP kinase in prolactin responsive mammary cells. *Breast Cancer Res Treat* **40**: 141–149

Di Leo A, Ferrari L, Bajetta E, Bartoli C, Vicario G, Moglia D, Miceli R, Callegari M and Bono A (1995) Biological and clinical evaluation of lanreotide (BIM 23014), a somatostatin analogue, in the treatment of advanced breast cancer. A pilot study by the I.T.M.O. group. Italian trials in medical oncology. *Breast Cancer Res Treat* **34**: 237–244

Erwin RA, Kirken RA and Malabarba MG (1995) Prolactin activates ras via signalling proteins SHC, growth factor receptor bound 2, and son of sevenless. *Endocrinology* **136**: 3512–3518

Evans AA, Crook T, Laws SAM, Gough AC, Royle GT and Primrose JN (1997) Analysis of somatostatin receptor subtype mRNA expression in human breast cancer. *Br J Cancer* **75**: 798–803

© Cancer Research Campaign 1999

Folkman J (1995) Clinical applications of research on angiogenesis. *N Engl J Med* **333**: 1757–1763

Goldhirsch A and Gelber RD (1996) Endocrine therapies of breast cancer. *Semin Oncol* **23**: 494–505

Gonzalez-Barcena D, Molina-Ayala MA, Cortez-Morales A, Vadillo-Buenfil M, Cardenas-Cornejo I, Comaru-Schally AM and Schally AV (1998) Response to administration of somatostatin analog RC-160 (vapreotide) in patients with advanced prostatic cancer at the relapse time. Proc 80th Annual Meeting Endocrine Soc, pp 421, abstract 164

Hankinson SE, Willett WC, Colditz GA, Hunter DJ, Michaud DS, Deroo B, Rosner B, Speizer FE and Pollak M (1998) Circulating concentrations of insulin-like growth factor-I and risk of breast cancer. *Lancet* **351**: 1393–1396

Hayes DF, Henderson IC and Shapiro CL (1995) Treatment of metastatic breast cancer: present and future prospects. *Semin Oncol* **22** (suppl. 5): 5–21

Helle SI, Geisler J, Poulsen JP, Hestdal K, Meadows K, Collins W, Tveit KM, Viste A, Holly JMP and Lonning PE (1998) Microencapsulated octreotide pamoate in advanced gastrointestinal and pancreatic cancer: a phase I study. *Br J Cancer* **78**: 14–20

Helle SI and Lonning PE (1996) Insulin-like growth factors in breast cancer. *Acta Oncol* **35**(suppl. 5): 19–22

Hofland LJ, van Koetsfeld PM, Waajers M, Zuyderwijk J and Lamberts SWJ (1994) Relative potencies of the somatostatin analogs octreotide, BIM-23014, and RC-160 on the inhibition of hormone release by cultured human endocrine tumor cells and normal rat anterior pituitary cells. *Endocrinology* **134**: 301–306

Holly J (1998) Insulin-like growth factor-I and new opportunities for cancer prevention. *Lancet* **351**: 1373–1375

Ingle JN, Kardinal CG, Suman VJ, Krook JE and Hatfield AK (1996) Octreotide as first-line treatment for women with metastatic breast cancer. *Invest New Drugs* **14**: 235–237

Klijn JGM, Berns PMJJ and Foekens JA (1993) Prognostic factors and response to therapy in breast cancer. *Cancer Surv* **18**: 165–198

Lamberts SWJ, Krenning EP and Reubi J (1991) The role of somatostatin and its analogs in the diagnosis and treatment of tumors. *Endocrine Rev* **12**: 450–482

Lee JM, Erlich RB, Bruckner HW, Szrajer L and Ohnuma T (1993) A somatostatin analogue (SMS 201-995) alters the toxicity of 5-fluorouracil in Swiss mice. *Anticancer Res* **13**: 1453–1456

Liebow C, Reilly C, Serrano M and Schally AV (1988) Somatostatin analogs inhibit growth of pancreatic cancer by stimulating tyrosine phosphatase. *Proc Natl Acad Sci USA* **85**: 1–22

Macaulay VM (1992) Insulin-like growth factors and cancer. *Br J Cancer* **65**: 311–320

Manni A, Boucher AE, Demers LM, Harvey HA, Lipton A, Symmonds MA and Bartholomew M (1989) Endocrine effects of combined somatostatin analog and bromocriptine therapy in women with advanced breast cancer. *Breast Cancer Res Treat* **14**: 289–298

Morere JF, Cour V, Breau JL, Boaziz C, Basin C and Israel L (1989) Stabilising effect of BIM23014, a long acting somatostatin analog in 30 cases of advanced breast cancer: a phase II study. *Proc Am Soc Clin Oncol* **8**: 47 (abstract 179)

Nagy A, Schally AV, Halmos G, Armatis P, Cai R-Z, Csernus V, Kovacs M, Koppan M, Szepeshazi K and Kahan Z (1998) Synthesis and biological evaluation of cytotoxic analogs of somatostatin containing doxorubicin or its intensely potent derivative, 2-pyrrolinodoxorubicin. *Proc Natl Acad Sci USA* **95**: 1794–1799

Patel PC, Barrie R, Hill N, Landeck S, Kurozawa D and Woltering EA (1994) Postreceptor signal transduction mechanisms involved in octreotide induced inhibition of angiogenesis. *Surgery* **116**: 1148–1152

Pinski J, Schally AV, Halmos G, Szepeshazi K, Groot K, O'Byrne KJ and Cai R-Z (1994) Effects of somatostatin analogue RC-160 and bombesin/gastrin-releasing peptide antagonist on the growth of human small-cell and non-small-cell lung carcinomas in nude mice. *Br J Cancer* **70**: 886–892

Pinski J, Schally AV, Halmos G, Szepeshazi K and Groot K (1996) Somatostatin analog RC-160 inhibits the growth of human osteosarcomas in nude mice. *Int J Cancer* **65**: 870–874

Pollak M (1996) Enhancement of the anti-neoplastic effects of tamoxifen by somatostatin analogues. *Digestion* **57** (suppl 1): 29–33

Pollak M and Schally AV (1998) Mechanisms of antineoplastic action of somatostatin analogs. *Proc Soc Exp Biol Med* **217**: 143–152

Poston GJ and Schally AV (1993) Somatostatin analogs and pancreatic cancer. *Int J Pancreatology* **14**: 64–66

Prevost G and Israel L (1993) Somatostatin and somatostatin analogues in human breast carcinoma. *Recent Results Cancer Res* **129**: 63–70

Qin Y, Eftl T, Groot K, Horvath J, Cai R-Z and Schally AV (1995) Somatostatin analog RC-160 inhibits growth of CFPAC-1 human pancreatic cancer cells in vitro and intracellular production of cyclic adenosine monophosphate. *Int J Cancer* **60**: 694–700

Reichlin S (1983*a*) Somatostatin (First of two parts). *N Engl J Med* **309**: 1495–1501

Reichlin S (1983*b*) Somatostatin (Second of two parts). *N Engl J Med* **309**: 1556–1563

Schally AV (1988) Oncological applications of somatostatin analogues. *Cancer Res* **48**: 6977–6985

Setyano-Han B, Henkelman MS, Foekens JA and Klijn JGM (1987) Direct inhibitory effects of somatostatin (analogues) on the growth of human breast cancer cells. *Cancer Res* **47**: 1566–1570

Srkalovic G, Szende B, Redding TW, Groot K and Schally AV (1989) Receptors for D-Trp6-luteinizing hormone-releasing hormone, somatostatin, and insulin-like growth factor I in MXT mouse mammary carcinoma (42987). *Proc Soc Exp Biol Med* **192**: 209–218

Stolfi R, Parisi AM, Natoli C and Iacobelli S (1990) Advanced breast cancer: response to somatostatin. *Anticancer Res* **10**: 203–204

Szende B, Lapis K, Redding TW, Srkalovic G and Schally AV (1989) Growth inhibition of MXT mammary carcinoma by enhancing programmed cell death (apoptosis) with analogs of LH-RH and somatostatin. *Breast Cancer Treat Res* **14**: 307–314

Szende B, Schally AV and Lapis K (1991) Immunocytochemical demonstration of tissue transglutaminase indicative of programmed cell death (apoptosis) in hormone sensitive mammary tumours. *Acta Morphol Hung* **39**: 53–58

Szepeshazi K, Lapis K and Schally AV (1991) Effect of combination treatment with analogs of luteinizing hormone-releasing hormone (LH-RH) or somatostatin and 5-fluorouracil on pancreatic cancer in hamsters. *Int J Cancer* **49**: 260–266

Szepeshazi K, Milovanovic S, Lapis K, Groot K and Schally AV (1992) Growth inhibition of oestrogen independent MXT mouse mammary carcinomas in mice treated with an agonist or antagonist of LH-RH, an analog of somatostatin, or a combination. *Breast Cancer Res Treat* **21**: 181–192

Teicher BA, Holden SA, Ara G, Sotomayor EA, Huang ZD, Chen YN and Brem H (1994) Potentiation of cytotoxic cancer therapies by TNP470 alone and with other anti-angiogenic agents. *Int J Cancer* **57**: 920–925

Tornell J, Carlsson B, Pohjanen P, Wennbo H, Rymo L and Isaksson OGP (1992) High frequency of mammary adenocarcinomas in metallothionein promoter-human growth hormone transgenic mice created from two different strains of mice. *J Steroid Biochem Mol Biol* **43**: 237–242

Van Eijck CHJ, Krenning EP, Bootsma A, Oei HY, Van Pel R, Lindesmans J, Jeekel J, Reubi J-C and Lamberts SWJ (1994) Somatostatin-receptor scintigraphy in primary breast cancer. *Lancet* **343**: 640–643

Vennin PH, Peyrat JP, Bonneterre J, Louchez MM, Harris AG and Demaille A (1989) Effect of the long-acting somatostatin analogue SMS 201-995 (Sandostatin) in advanced breast cancer. *Anticancer Res* **9**: 153–156

Weckbecker G, Tolcsvai L, Stolz B, Pollak M and Bruns C (1994) Somatostatin analogue octreotide enhances the antineoplastic effects of tamoxifen and ovariectomy on 7,12-dimethylbenz(*a*)anthracene-induced rat mammary carcinoma. *Cancer Res* **54**: 6334–6337

Weckbecker G, Raulf F, Tolcsvai L and Bruns C (1996) Potentiation of the anti-proliferative effects of anti-cancer drugs by octreotide in vitro and in vivo. *Digestion* **57** (Suppl. 1): 22–28

Xu Y, Song J, Berelowitz M and Bruno JF (1996) Estrogen regulates somatostatin receptor subtype 2 messenger ribonucleic acid expression in human breast cancer cells. *Endocrinology* **137**: 5634–5640

*© Cancer Research Campaign 1999*
